# Design and Implementation of an Event-Driven Smart Sensor Node for Wireless Monitoring Systems

**DOI:** 10.3390/s23249737

**Published:** 2023-12-10

**Authors:** Changrong Liu, Junjie Yang, Zhenghao Lu, Changnan Chen, Jiachou Wang, Dacheng Xu, Xinxin Li

**Affiliations:** 1 School of Electronic and Information Engineering, Soochow University, Suzhou 215006, China; 2 State Key Laboratory of Transducer Technology, Shanghai Institute of Microsystem and Information Technology, Chinese Academy of Sciences, Shanghai 200050, China

**Keywords:** event-driven, piezoelectric energy harvesting, wireless sensor node, smart sensor, wireless monitoring system, wireless communication

## Abstract

In this paper, an event-driven wireless sensor node is proposed and demonstrated. The primary design objective is to devise a wireless sensor node with miniaturization, integration, and high-accuracy recognition ability. The proposed wireless sensor node integrates two vibration-threshold-triggered energy harvesters that sense and power a threshold voltage control circuit for power management, a microcontroller unit (MCU) for system control, a one-dimensional convolutional neural network (1D-CNN) environment data analysis and vibration events distribution, and a radio frequency (RF) digital baseband transmitter with IEEE 802.15.4-/.6 protocols. The dimensions of the wireless sensor node are 4 × 2 × 1 cm^3^. Finally, the proposed wireless sensor node was fabricated and tested. The alarming time for detecting the vibration event is less than 6 s. The measured recognition accuracy of three events (knock, shake, and heat) is over 97.5%. The experimental results showed that the proposed integrated wireless sensor node is very suitable for wireless environmental monitoring systems.

## 1. Introduction

Remote wireless sensors for gathering important information, distinguishing events, and transmitting vital data of analysis are in great demand in the area of environmental monitoring such as oil pipelines, and fences [1,2,3,4,5,6]. Currently, most wireless sensors are “not smart” in that they continuously collect and transmit data. As a result, these wireless sensors require a constant power supply and the sensors can only operate for a limited amount of time because of the energy limitations of the sensor battery. To solve the sensor power supply issue, many kinds of self-powered sensors have been proposed that use energy resources such as wind [3], vibration [7], light [8,9,10], thermal [11], electromagnetic [12], breaking [13], rotation motion [14], etc. A framework to model and characterize the area for multiple-source energy-harvesting systems was proposed in [15]. Generating energy from ambient environments to power the wireless sensor nodes has become attractive.

In these ambient environments, vibration energy is ubiquitously distributed in the industrial environment. Consequently, the piezoelectric vibration energy harvester can be utilized to convert vibration energy into electrical energy and can sense environmental vibration information. When the piezoelectric energy harvester is used to detect environmental vibration, the power consumption related to the sensor can be saved. In addition, in specific applications for the safety monitoring of field fences, oil pipelines, etc., using the “event-driven” principle, we only focus on whether a sabotage event occurs [16,17,18,19,20,21].

As for “event-driven” sensing systems, they usually first rely on domain knowledge to extract the characteristics of the voltage signal, use the extracted characteristics to interpret the signal, and then use these characteristics to identify different environmental vibration modes [22,23,24,25,26,27,28,29]. In [22], the potential of human activity recognition from kinetic energy harvesting (HARKE) was quantified, with an accuracy of 80 to 95 percent. KEH-Gait was proposed in [23] to achieve comparable recognition accuracy when multiple steps are used and reduce energy consumption by 82.15 percent. In [24], a novel architecture for simultaneous energy harvesting and gait recognition detects human gait with 12% higher accuracy compared to the state of the art. Nevertheless, environmental vibration is complex, and the voltage characteristics generated by the energy harvester are always caused by many factors. Only considering some of these factors and establishing assumptions may cause low recognition accuracy. 

Typically, wireless sensor nodes support one communication protocol. However, with the demands of data capacity and transmission distance, there is a contradiction between a high data rate and long-range communication. The SimpliciTi protocol that directly runs on CC1110 chips as the communication protocol between the sensor node and the router node was adopted by [19]. IEEE 802.15.4 was chosen as the communication protocol in [30,31], as an IEEE 802.15.4 radio enabling up to 100 m outdoor communication range (single hop). Moreover, the wireless body area networks (WBAN, IEEE 802.15.6) nodes used for sensing purposes have gained remarkable achievement [32,33]. A self-powered wireless temperature sensor node for power grid monitoring was proposed in [34], where the communication distance was 70 m with 2-FSK modulation on the carrier frequency of 433 MHz. 

To take on the above-mentioned challenges, this paper presents a fully smart wireless sensor node (WSN) with the abilities of sensing, powering, computing, and communication, which is a complete wireless sensor module comprising two energy harvesters (EH), a threshold voltage control circuit for power management, a microcontroller (MCU), and a radio frequency (RF) digital baseband transmitter. The integrations of sensing and powering, information detection and event recognition, long-distance communication, and high transmission rate are the main goals of this study.

## 2. Description of the Smart Wireless Sensor Node

**Figure 1:**
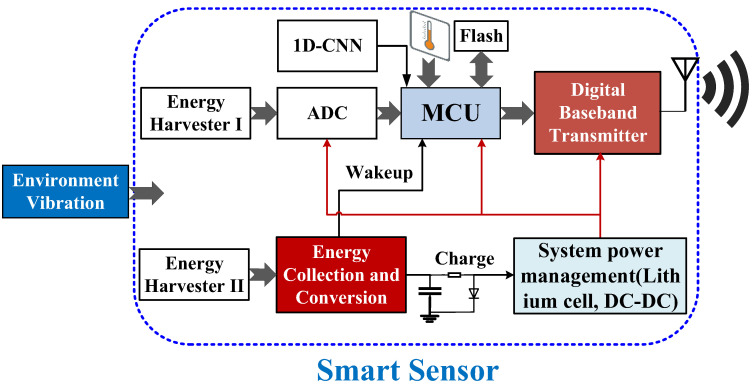
Block architecture of the wireless smart sensor system.

Figure 1 shows the block architecture of the smart WSN system. The proposed WSN system consists of five main parts: the piezoelectric energy harvesters (PEHs) for sensing and powering, a threshold voltage control circuit for power management and wakeup, an MCU for system control and a one-dimensional convolutional neural network (1D-CNN) for calculation, an RF digital baseband transmitter with two communication protocols, and a planar transmit (Tx) antenna with compact size. A detailed description of the smart WSN system is depicted in Table 1. The purpose of introducing the temperature sensor is to consider this sensing node to be installed in the fence environment to simulate real-time alarms in case the fence encounters fire.

The major contributions of this system scheme can be summarized as follows:

(a) Two PEHs are utilized: one is for detecting the vibration activities and the other is for converting energy and power collection.
(b) The event recognition based on a lightweight 1D-CNN is calculated by an MCU. The proposed scheme can identify the event status in real time and send the data of interest selectively to reduce RF power consumption.
(c) An RF digital baseband transmitter with two communication protocols was adopted in this study. The WSN can select the communication protocol with different data rates and communication distances.
(d) A fully WSN system with compact size was designed and implemented and the proposed WSN was tested to evaluate the system performance.

## 3. Design of the Smart Sensor and Measurement

### 3.1. Energy Harvester

Piezoelectric energy harvesters are widely utilized in the event-driven wireless sensor systems to sense environmental vibration information as they usually rely on the characteristics of the voltage signal, use the extracted characteristics to interpret the signal, and then identify different environmental vibration modes based on these characteristics. The PEH used in this study was developed in previous work [16,17,18,19]. 

**Figure 2:**
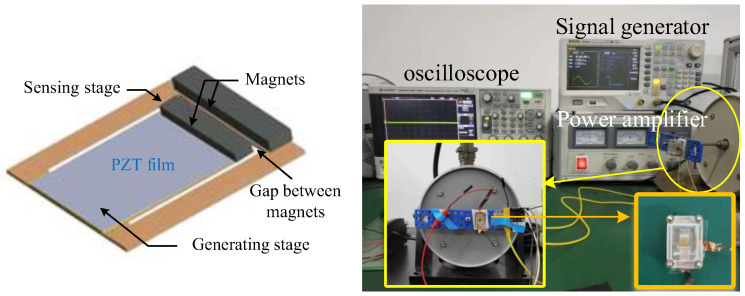
The proposed vibration-threshold-triggered energy harvester.

Figure 2 shows the structure of the proposed vibration-threshold-triggered energy harvester, where the energy harvester consists of two vibrating stages: the sensing stage and the generating stage. The photograph of the fabricated energy harvester and the measurement setup was described in Figure 2. A vibration table (JZK-5) was excited using a signal generator (Agilent 33120A, Agilent Technologies, Incorporated, Loveland, CO, USA) to generate sinusoidal oscillations. The energy collector that encapsulates the Plexiglas housing was secured to the vibration table. A standard accelerometer was installed near the energy collector to monitor the acceleration of vibrations. 

**Figure 3:**
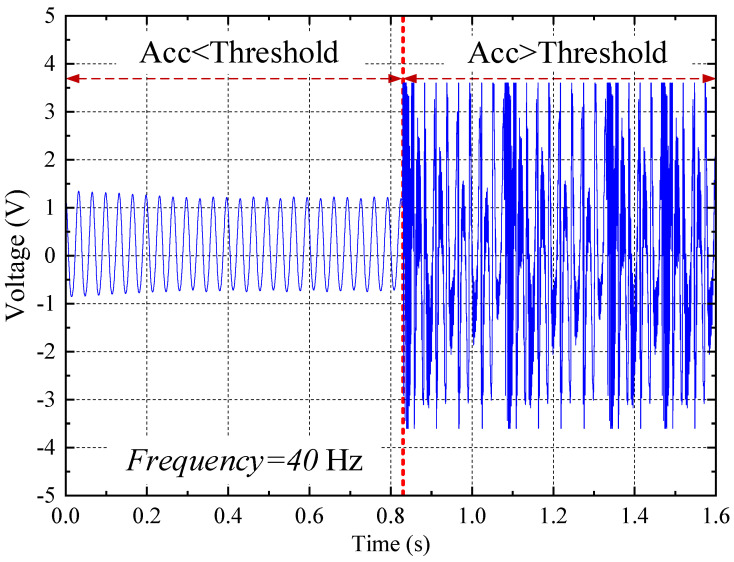
Measured results of the vibration-threshold-triggered energy harvester.

The measured result was depicted in Figure 3. It can be seen from Figure 3 that the output voltage is low when the input vibration acceleration is below the threshold; the output peak voltage can reach ~±3.6 V when the input vibration acceleration is over the threshold. Note that the resonant frequency of the PEH was designed to be close to 40 Hz to simulate practical environmental vibrations.

### 3.2. Vibration Event Recognition

PEHs are capable of simultaneously acquiring environmental vibration energy and information sense, and the design of vibration information sensing and pattern recognition systems based on this device is a research hotspot. Referring to the classical algorithm fully convolution network in the field of time series classification, a 1D-CNN is proposed to solve the problem of large computation and unable to extract multi-scale time features. 

Data collection: Three testers performed two types of damage to the fence by shaking and knocking at different frequencies and intensities, and collected the output voltage signals of the energy harvester in these two vibration modes. For the constructed 1600 samples, 70% is for training, and the rest is for testing.

**Figure 4:**
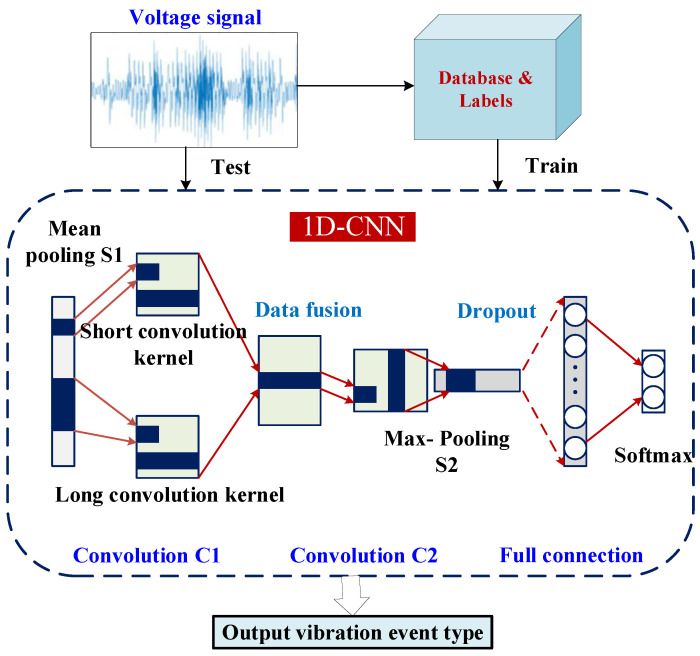
Architecture of vibration event recognition based on 1D-CNN.

Figure 4 shows the architecture of the proposed vibration event recognition based on 1D-CNN. Given the multi-scale time characteristics of the output voltage signal of the energy harvester, corresponding to the long-time scale (low-frequency) features and generation oscillator short time scale (high-frequency) features, the second convolution layer of the 1D-CNN model extracts features in parallel through the long and short convolution kernels.

The 1D-CNN model is suitable to achieve vibration pattern recognition with high accuracy while avoiding the problem of complicated feature extraction. The network has fewer parameters and is easy to deploy on a microprocessor. After optimization, the average pooling window in the first layer is 5, the second layer of long and short convolution kernel width is (10, 2), and the two layers of the convolutional layer under the parameter design with the number of filters are (8, 16); the 1D-CNN model can achieve a classification accuracy of 95.2%.

**Figure 5:**
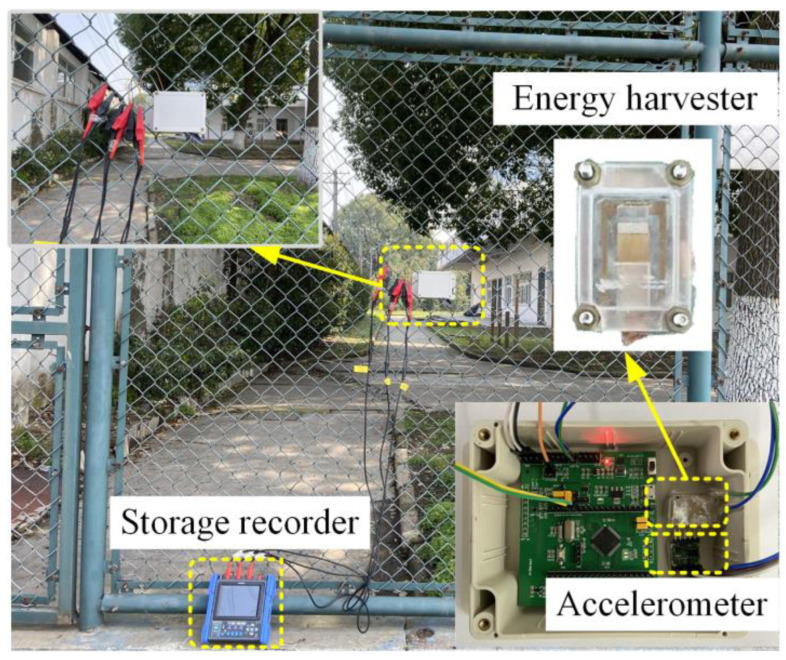
Measurement setup of the vibration event recognition.

Finally, a complete system was built and tested as shown in Figure 5. The system recognition result was output by the two general-purpose input/output (GPIO) ports of the MCU, and the real-time output of the GPIO ports was measured by the memory recorder (MR8880) to analyze the current recognition results. To test the “event-driven” mechanism of the system, the storage recorder records the output of the clamp circuit at the same time and compares and analyzes the working process of the system with the recognition result. During the test, the fence will be continuously knocked or shaken for a long period, and the recognition accuracy of the system during this period will be calculated. The actual recognition accuracy of knocking was 95.0%, the actual recognition accuracy of shaking was 97.5%, and the overall recognition accuracy was 96.2%. The measured results show that when the acceleration generated by shaking or knocking on the fence reaches the preset threshold of the PEH, the MCU will wake up in 0.1 s and output the vibration event recognition results in 0.9 s, and the measured actual recognition accuracy of the system reaches 96.2%. 

### 3.3. Energy Collection and Conversion

For the realization of passive or low-power WSNs, it is extremely important to maximize the capture of the electrical energy output by the PEH. The core part of the energy supply sub-system is the energy collection and conversion circuit, as it collects the vibration energy from the environment, and converts it into the electric energy for the WSN. The subsystem can wake up the control circuit and charge the super-capacitor, thus extending the lifetime of the WSNs.

**Figure 6:**
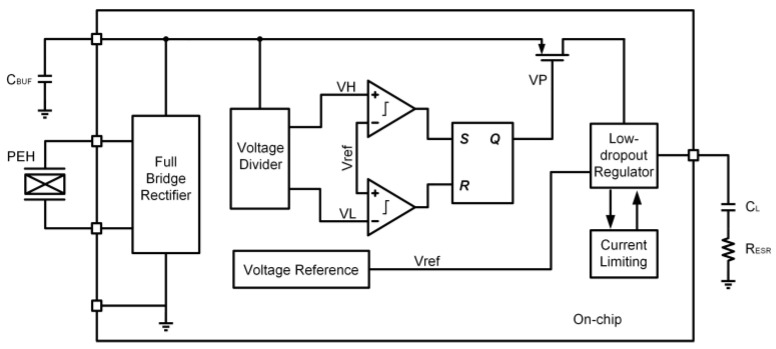
Block diagram of energy-harvesting chip.

Figure 6 shows the block diagram of the energy-harvesting chip. The proposed energy-harvesting chip mainly includes four modules: rectifier bridge, dual-threshold management circuit, reference source, and Low Drop Out (LDO) Regulator. The rectifier bridge is a full-wave rectifier to convert the AC signal into a DC signal; the dual-threshold management circuit controls the charging and discharging of the energy storage capacitor; the reference source provides a reference voltage independent of temperature, power supply voltage, and process and a reference current with a specific temperature coefficient; and LDO provides a stable output voltage with driving capability.

**Figure 7:**
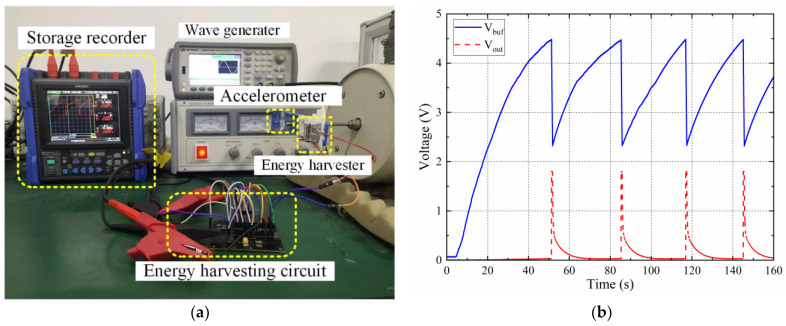
Measurement of the energy-harvesting chip: (**a**) measurement setup, (**b**) measured results (V_buf_: voltage of the capacitor during the charge; V_out_: voltage of the LDO during the discharge).

To verify the design concept, the proposed energy-harvesting chip was fabricated and measured. Figure 7a shows the measurement setup of the proposed energy harvesting chip. During the test, a 200 μF off-chip capacitor was used as the energy storage capacitor, and a 0.8 KΩ resistor was used instead of the load circuit. 

Figure 7b depicts the working process of the energy-harvesting chip. The dual-threshold management circuit always detects the voltage V_buf_ on the energy storage capacitor. When the energy storage capacitor starts to charge, V_buf_ rises from zero to VH (4.4 V). The dual-threshold management circuit turns on the switch. At this time, the energy storage capacitor supplies power to the LDO, and the LDO is normal. The work outputs a stable voltage with driving capability. When the voltage V_buf_ on the energy storage capacitor drops to VL (2.3 V), the dual-threshold management circuit turns off the switch, the LDO stops working, and the energy storage capacitor continues to charge. During the discharge of the energy storage capacitor, the output voltage of the LDO can be stabilized at 1.8 V.

### 3.4. Digital Baseband Transmitter

**Figure 8:**
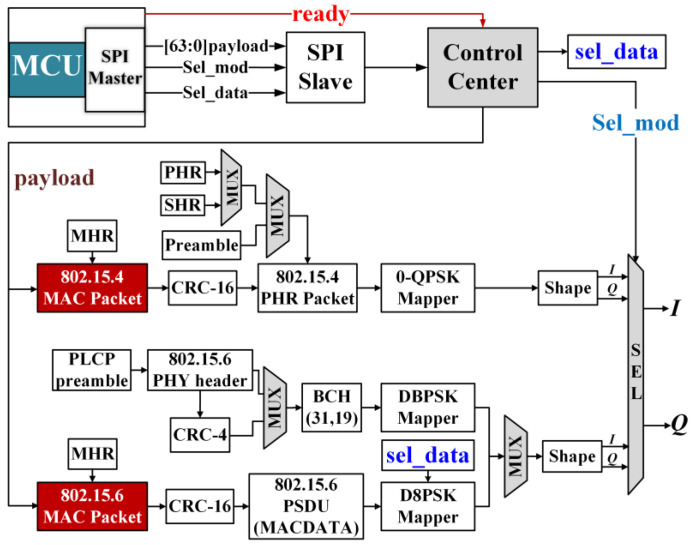
Proposed digital baseband with IEEE 802.15.4/.6 protocols.

To achieve the integration of long communication distance and high data rate, two protocols of IEEE 802.15.4/6 are adopted. The initialization settings will be written into the control register files of the MCU through the SPI bus, and then the baseband chip will be initialized according to the written values. SPI generally supports higher data rates compared to I2C and UART. The complete baseband transmission process is shown in Figure 8. 

In this design, the ready signal is used to control the working status of each module. The payload signal is the data to be sent. When the MCU sends out the start signal, one of the two protocols will be selected according to the value of the Sel_mod signal, and the payload data are sent to the corresponding MAC Packet module for the MAC layer grouping. After selecting the corresponding protocol, the other protocol channel will be closed. All the data will be sent to the packetizer together with MAC. The modulated data are shaped and filtered to obtain the IQ dual-channel baseband digital modulated signal, which is connected to the DAC module of the RF part.

**Figure 9:**
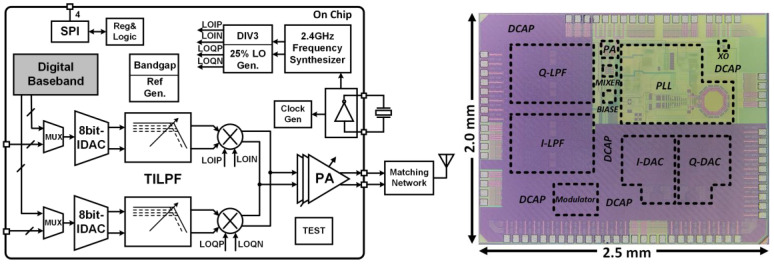
Proposed digital baseband transmitter.

Besides the digital baseband, a transmitter (Tx) integrated chip (IC) is desired to transmit the data. Figure 9 shows the overall block diagram of the Tx IC. According to the main functions, the transmitter chip can be divided into five parts: bias and clock generation circuit, transmitter radio frequency circuit, transmitter intermediate frequency circuit, digital baseband circuit, and test circuit.

The entire digital baseband transmitter was integrated and fabricated. In the OQPSK and D8PSK test modes, the symbol rates are 500 ksps and 750 ksps, respectively. The measured results demonstrated that the proposed IC of IEEE 802.15.4/6 can work well, which is suitable for WSN applications. The output power of the Tx chip can be controlled by changing the gain of the power amplifier (PA). The maximum output power level of the Tx chip is 3 dBm.

## 4. System Design and Measurement

### 4.1. System Design

**Figure 10:**
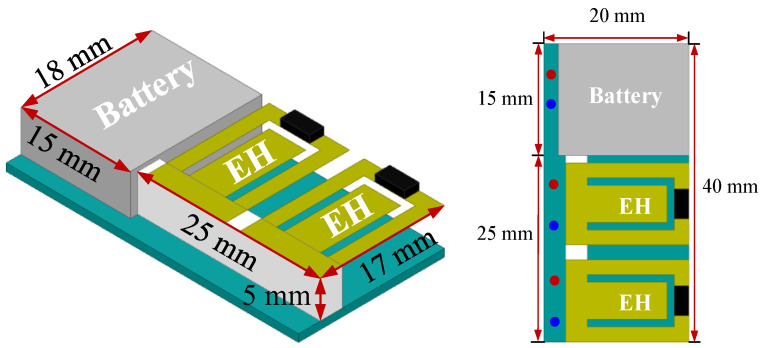
Fully WSN system design.

Figure 10 shows the fully WSN system design, where the system size including the printed circuit board (PCB) is less than 4 × 2 × 1 cm^3^ (length × width × height). The two PEHs were utilized for powering and sensing. And the battery is for the power supply. The dimensions of the battery are 17 × 15 × 5 mm^3^ with a capacity of 3.7 V, 150 mAh. The battery cell and two PEHs were all glued on the backside of the PCB. 

**Figure 11:**
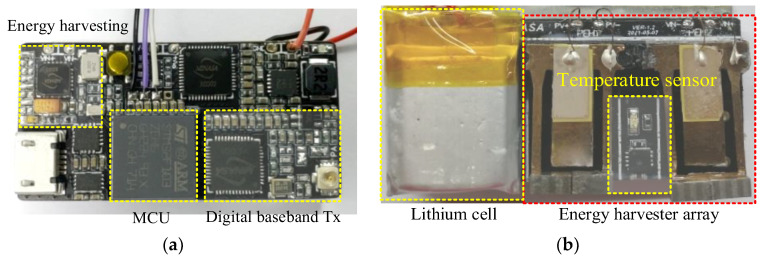
Photograph of the proposed WSN system design: (**a**) top view, (**b**) bottom view.

The photograph of the proposed WSN was shown in Figure 11. It can be seen that the PCB includes most of the above-mentioned subsystems and a temperature sensor was between the two PEHs for environment temperature capture. Note that the Tx antenna was not included in the system design and it can be designed based on the system testing environment. An RF Ipex connector with small size and low profile was soldered on the edge of the PCB, which can be terminated with mini coaxial cables.

### 4.2. Tx Antenna Design

**Figure 12:**
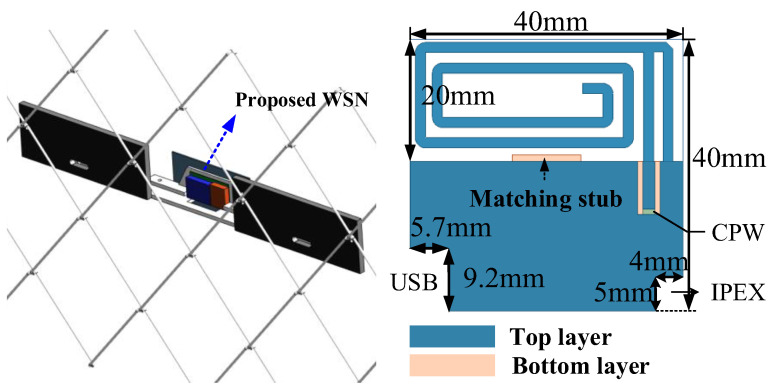
Tx antenna design.

After considering the system design, a Tx antenna with compact size and low profile for the WSN is desired. Note the testing environment should be considered for the antenna design as shown in Figure 12, where the proposed WSN was fixed to the fence and reinforced by aluminum material. Based on electromagnetic theory, it is seen that the planar inverted-F antenna cannot close to the ground plane, as the ground plane will cause an opposite current that decreases the radiation efficiency. In this condition, the main radiator was over the WSN system board to have open space, thus achieving acceptable radiation performance. Also, the raised USB interface and the Ipex connector should be considered for the further package; two rectangular places were cut on the antenna. The proposed Tx antenna was designed on an FR4 substructure with a thickness of only 0.6 mm. Ansys HFSS was utilized for the antenna simulation. The dimensions of the Tx antenna are 40 × 40 × 0.6 mm^3^, and the entire volume of the smart WSN is <8 cm^3^.

The measured results show that the proposed Tx antenna can operate in the frequency range of 416~421 MHz for |S11| < −6 dB. The measured peak gain at 416 MHz is about −14 dBi, which was mainly caused by the small aperture area. The calculated gain is ~−13 dBi, which is in good agreement with the measured one. The radiation pattern of the Tx antenna is omnidirectional radiation and is very suitable for wireless sensor nodes.

Besides the Tx antenna, a receive antenna is needed to receive the transmitted signal from the WSN. A high-gain circularly polarization (CP) antenna was proposed. The CP antenna can effectively avoid the polarization mismatching between the transmitter and the receiver. The measured impedance bandwidth is from 390 to 420 MHz for |S11| < −10 dB. The measured axial ratio (AR) bandwidth is in the frequency range of 393–423 MHz for AR < 3 dB. And the measured peak gain is ~6.7 dBic at 416 MHz.

After the WSN was designed, the Tx and Rx antennas were tested, the link margin was analyzed, and the wireless communication testing should be conducted to evaluate the performance of the subsystems, the event recognition rate, and the communication ability.

The transmitted signal from the proposed WSN will be received by the high-gain CP Rx antenna, and the received signal will be amplified by an LNA and sent to the Universal Software Radio Peripheral (USRP) N210 (https://www.ettus.com/all-products/UN210-KIT/) (30 September 2021) and demodulated by the GNU radio 3.9.3.0(2021-09-30) software platform. Based on the demodulation results of the GNU radio, a software interface aspect was designed to view the recognition results easily.

### 4.3. Wireless Communication Testing

**Figure 13:**
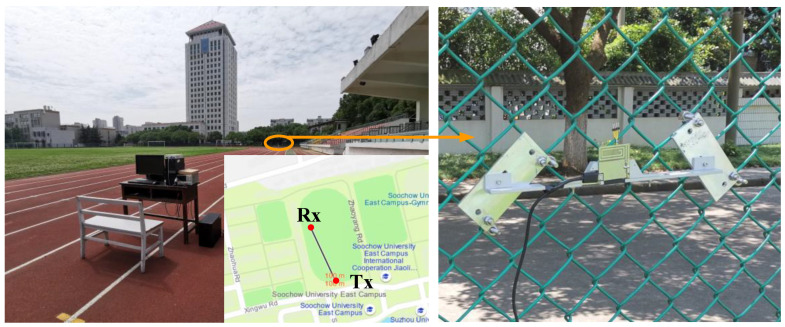
Wireless communication measurement setup.

Figure 13 shows the Bing map of the measurement environment, which is located on the stadium of Soochow University East Campus. The WSN was fixed on a protective fence of the stadium. And the distance between the WSN and the receiver is over 100 m. The proposed event-driven smart WSN was tested under different events and communication protocols. In the testing, the fence was knocked by a hammer and was shaken by a human hand to simulate the “knock” and “shake” excitation, respectively. And the “heat” excitation was simulated by a hot air gun. Each test needs to be repeated 120 times. As for the heat testing, the WSN temperature was reduced to the room temperature level before the retest.

**Figure 14:**
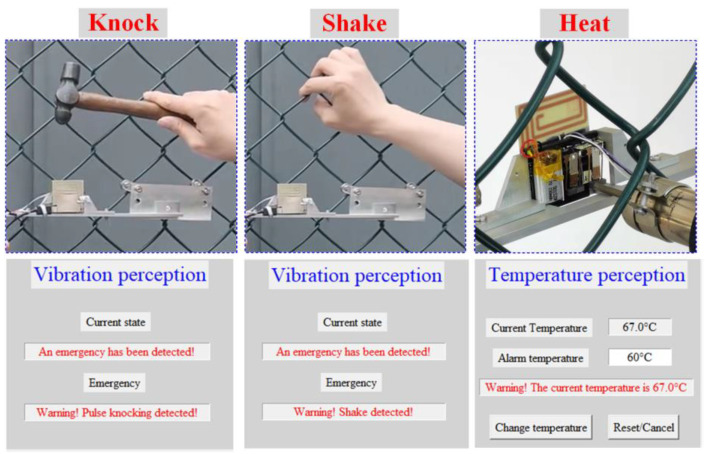
Output recognition results shown on the receiver.

The detailed recognition results of the wireless communication testing were shown in Figure 14. Finally, the recognition results were summarized and listed in Table 2. The final recognition accuracy was >97.5% in the three-event cases, which demonstrates that the proposed WSN was very suitable for event-driven wireless monitoring applications.

### 4.4. Power Consumption Discussion

Besides the performance testing, the power consumption should also be discussed as it affects the lifetime of the WSN. The details of the power consumption of subsystems are listed in Table 3, including quiescent current and dynamic current. It can be seen that the main power consumption was caused by the MCU and the Tx chip. The power from the MCU is primarily needed for 1D-CNN calculation. And the second power consumption comes from the Tx chip, which is reasonable for the WSN systems. When the WSN was set as continuous transmitting mode, the power consumption of the entire WSN is 173.25 mW with a dynamic current of 52.5 mA. In practice, the alarms of interest will be less, and the average power consumption will be very low.

Referring to [35], the average current consumption is given by:

$$I_{avg}=\frac{t_{active}}{T_{total}}I_{active}+(1-\frac{t_{active}}{T_{total}})I_{sleep}$$

where *I_active_* and *t_active_* are the active current and active transmission time of the device, respectively. The *I_sleep_* is the sleep current. In this manuscript, the knocking, shaking, and heating are tested 120 times in one day, and their test time lasted 5.2 s, 5.0 s, 3.7 s, respectively. The rest time was sleep time, and the sleep currents are shown in Table 3.

$$\begin{array}{l} t_{active}=1688\;s\\ T_{total}=86,400\;s\\ I_{active}=52.5\;mA\\ I_{sleep}=4.5\;\mu A\\ I_{avg}=\frac{t_{active}}{T_{total}}I_{active}+(1-\frac{t_{active}}{T_{total}})I_{sleep}\\ \approx 1.03\;mA \end{array}$$

**Figure 15:**
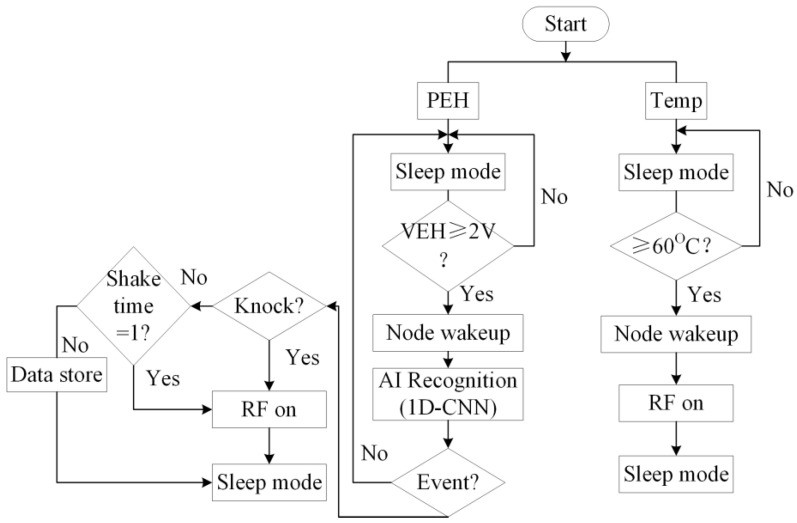
Operation flowchart of the WSN.

To further reduce the average power consumption, an operation flowchart of the WSN is presented in Figure 15. When the output voltage of the energy harvester (VEH) is over 2 V, the MCU will start to analyze the event and distribute the event type and the WSN will start to work. If it is the event of interest, the RF will be turned on and the WSN will be switched to the sleep mode. The operation flow chart of the WSN can save the power and extend the lifetime of the WSN system. The powering can be carried out by collecting and converting the vibration energy.

## 5. Conclusions

An event-driven wireless sensor node with the abilities of sensing, powering, computing and communication was designed and implemented with miniaturization, integration, and high-accuracy recognition ability. The proposed wireless sensor node integrates two PEHs that can sense and power a threshold voltage control circuit for power management, an MCU for system control and 1D-CNN environment data analysis and vibration event distribution, and an RF digital baseband Tx with IEEE 802.15.4-/.6 protocols. The entire volume of the wireless sensor node is less than 8 cm^3^. 

Finally, the proposed wireless sensor node was fabricated and tested. The alarming time for detecting the vibration event is less than 6 s. The measured recognition accuracy of the WSN under three different events is over 97.5%. The results demonstrated that the proposed WSN has integrations of sensing and powering, information detection and event recognition, long-distance communication, and high transmission rate, which meet the requirements of wireless environmental monitoring applications such as fences.

Future work will focus on the distributed wireless sensor system design to further improve the recognition and reduce the power consumption.

## Figures and Tables

**Table 1:** Description of the wireless sensor node system.

Subsystem	Description
Energy harvester	Piezoelectric energy harvester
Temperature sensor	SHT30-DIS-B2.5kS
Energy collection and conversion	Average static power consumption: <1 µW (0.11 µm CMOS)
Analog-to-digital converter (ADC)	Ti ADS1015IRUGR
Microcontroller unit (MCU)	STM32F103ZEH6
Digital baseband transmitter	IEEE 802.15.4/-.6 based Transmitter (65 nm CMOS)
Power supply	Lithium cell (3.7 V 150 mAh)

**Table 2:** Recognition results of the WSN System.

Events	Number of Testing	Alarming Time	Recognition Rate
Knock	120	<5.2 s	97.5%
Shake	120	<5.0 s	98.3%
Heat	120	<3.7 s	100%

**Table 3:** Power consumption of the WSN.

Component	Voltage	Quiescent Current	Dynamic Power
ADS1015	3.3 V	0.5 μA	150 μA/0.5 mW
SHT30	2.5 V	0.2 μA	1.7 μA/4.25μW
MCU	2.5 V	3.8 μA	25 mA/62.5 mW
Tx chip	1.2 V	-	22.5 mA/27 mW
Energy harvesting chip	No power supply		

## Data Availability

Data are contained within the article.
